# Enhancing precision: A predictive model for ^177^Lu-DOTATATE treatment response in neuroendocrine tumors using quantitative ^68^Ga-DOTATATE PET and clinicopathological biomarkers

**DOI:** 10.7150/thno.98053

**Published:** 2024-06-11

**Authors:** Azadeh Akhavanallaf, Sonal Joshi, Arathi Mohan, Francis P Worden, John C Krauss, Habib Zaidi, Kirk Frey, Krithika Suresh, Yuni K Dewaraja, Ka Kit Wong

**Affiliations:** 1 Department of Radiology, University of Michigan, Ann Arbor, MI, USA.; 2 Division of Hematology and Oncology, Department of Internal Medicine, University of Michigan, Ann Arbor, MI, USA.; 3 Rogel Cancer Center, University of Michigan, Ann Arbor, MI, USA.; 4 Department of Biostatistics, University of Michigan, Ann Arbor, MI, USA.; 5 Division of Nuclear Medicine and Molecular Imaging, Geneva University Hospital, CH-1211 Geneva, Switzerland.; 6 Department of Nuclear Medicine and Molecular Imaging, University of Groningen, University Medical Center Groningen, 9700 RB Groningen, Netherlands.; 7 Department of Nuclear Medicine, University of Southern Denmark, DK-500, Odense, Denmark.; 8 University Research and Innovation Center, Óbuda University, Budapest, Hungary.

**Keywords:** SSTR-PET, images-based features, NET, PRRT, outcome prediction

## Abstract

**Purpose:** This study aims to elucidate the role of quantitative SSTR-PET metrics and clinicopathological biomarkers in the progression-free survival (PFS) and overall survival (OS) of neuroendocrine tumors (NETs) treated with peptide receptor radionuclide therapy (PRRT).

**Methods:** A retrospective analysis including 91 NET patients (M47/F44; age 66 years, range 34-90 years) who completed four cycles of standard ^177^Lu-DOTATATE was conducted. SSTR-avid tumors were segmented from pretherapy SSTR-PET images using a semiautomatic workflow with the tumors labeled based on the anatomical regions. Multiple image-based features including total and organ-specific tumor volume and SSTR density along with clinicopathological biomarkers including Ki-67, chromogranin A (CgA) and alkaline phosphatase (ALP) were analyzed with respect to the PRRT response.

**Results:** The median OS was 39.4 months (95% CI: 33.1-NA months), while the median PFS was 23.9 months (95% CI: 19.3-32.4 months). Total SSTR-avid tumor volume (HR = 3.6; P = 0.07) and bone tumor volume (HR = 1.5; P = 0.003) were associated with shorter OS. Also, total tumor volume (HR = 4.3; P = 0.01), liver tumor volume (HR = 1.8; P = 0.05) and bone tumor volume (HR = 1.4; P = 0.01) were associated with shorter PFS. Furthermore, the presence of large lesion volume with low SSTR uptake was correlated with worse OS (HR = 1.4; P = 0.03) and PFS (HR = 1.5; P = 0.003). Among the biomarkers, elevated baseline CgA and ALP showed a negative association with both OS (CgA: HR = 4.9; P = 0.003, ALP: HR = 52.6; P = 0.004) and PFS (CgA: HR = 4.2; P = 0.002, ALP: HR = 9.4; P = 0.06). Similarly, number of prior systemic treatments was associated with shorter OS (HR = 1.4; P = 0.003) and PFS (HR = 1.2; P = 0.05). Additionally, tumors originating from the midgut primary site demonstrated longer PFS, compared to the pancreas (HR = 1.6; P = 0.16), and those categorized as unknown primary (HR = 3.0; P = 0.002).

**Conclusion:** Image-based features such as SSTR-avid tumor volume, bone tumor involvement, and the presence of large tumors with low SSTR expression demonstrated significant predictive value for PFS, suggesting potential clinical utility in NETs management. Moreover, elevated CgA and ALP, along with an increased number of prior systemic treatments, emerged as significant factors associated with worse PRRT outcomes.

## Introduction

Neuroendocrine neoplasms are heterogeneous tumors originating from neuroendocrine cells in various organs and include both well-differentiated neuroendocrine tumors (NETs) and poorly differentiated carcinomas. Approximately 20-50% of patients with NETs have metastatic disease at the time of diagnosis without an available curative surgical option, necessitating systemic treatment 1, 2.

The majority of well-differentiated NETs overexpress somatostatin receptors (SSTRs), which present a target for theragnostic radiopharmaceuticals. Treatment with radiolabeled-conjugated somatostatin analogs, called peptide receptor radionuclide therapy (PRRT) was introduced using ^111^In-pentetreotide and was extended by developing ^90^Y-octreotide and ^177^Lu-DOTATATE treatments 3. Upon the approval of ^177^Lu-DOTATATE on the basis of the phase III NETTER-1 trial 4, ^177^Lu-PRRT has been widely used in the management of advanced well-differentiated NETs. Despite the improved progression-free survival (PFS) rate from ^177^Lu-DOTATATE, PRRT continuous to be a palliative approach, as the objective response rate has been reported about 30% 5, 6. Therefore, appropriate sequencing of PRRT and selection of optimal candidates for this therapy remains an area of ongoing research. Moreover, potential toxicities and the high cost of PRRT warrant further investigation to optimize PRRT.

Currently, patient eligibility for PRRT is based on qualitative assessment of SSTR expression (Krenning score 3-4), visualized with one of the approved radiopharmaceuticals (^68^Ga-DOTATATE, ^68^Ga-DOTATOC, and ^64^Cu-DOTATATE) using positron emission tomography (PET) 7. Several studies investigated the predictive value of baseline SSTR-PET on PRRT outcome. Multiple quantitative metrics derived from SSTR-PET images, such as SSTR density, tumor burden and localization, as well as tumor size and heterogeneity have been reported to be predictive for NET patients who underwent PRRT 8-10. Several clinicopathological biomarkers have also been proposed as predictive for PRRT outcome including type of primary tumor, proliferation index, previous systemic therapy, chromogranin A (CgA) and blood-based genomic biomarkers 1, 11, 12. To date, however, the predictors of PRRT outcome have not been thoroughly elucidated.

The aim of this study is to identify clinical parameters based on SSTR-PET and clinicopathological biomarkers that predict PRRT response in terms of overall survival (OS) and progression-free survival (PFS) for NETs. This work makes a multifaceted contribution to the field: 1. SSTR-avid total body tumor volume labeled with anatomical location was segmented using a semi-automatic workflow and verified by a clinician. 2. A comprehensive set of predictors including image-based features and biomarkers were incorporated in the analysis.

## Materials and Methods

### Patient Population

**Figure 1:**
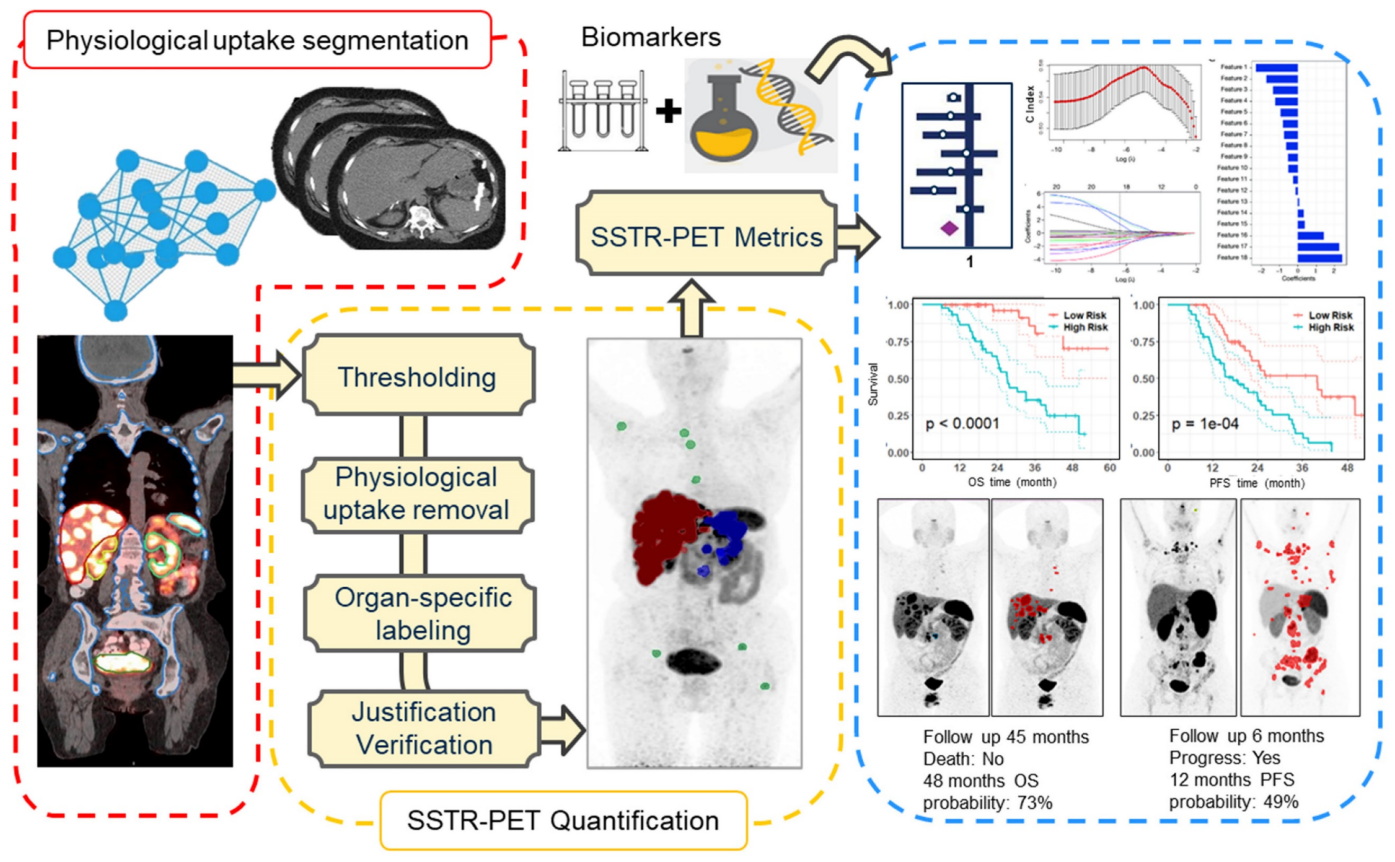
left panel: deep learning based physiological uptake segmentation, middle panel: quantification of SSTR-PET images and right panel: survival analysis.

In a retrospective, single-center study conducted at the University of Michigan, we assessed a group of 180 patients who underwent at least one cycle of ^177^Lu-DOTATATE treatment between 2018 and 2021 in routine clinical practice. This study was approved by the local Institutional Review Board and informed consent was obtained from participants. From this initial cohort, we excluded 89 patients based on specific criteria: (1) use of PET tracer other than ^68^Ga-DOTATATE; (2) those who did not complete four treatment cycles; (3) other tumors (paraganglioma, neuroblastoma); (4) patients with other type of cancers at the time of therapy; (5) missing SSTR-PET; (6) insufficient follow-up period, defined as not having at least one clinical visit after completing their treatment. Consequently, a total of 91 patients with histologically proven NETs who completed four cycles of the approved protocol for ^177^Lu-PRRT (7.4 GBq infusions; ~2-month intervals) met the inclusion criteria. As of the data lock in September 2023, 35 patients (38%) had succumbed to their condition, and 60 patients (66%) had exhibited disease progression (Supplemental-Figure 1).

### SSTR-PET/CT Imaging and PET-derived Metrics

Patient eligibility required PET scans to be acquired less than six months prior to the planned PRRT. ^68^Ga-PET/CTs were performed on a Biograph mCT (Siemens Healthineers) (84/91), Biograph TruePoint (Siemens Healthineers) (2/91), Discovery STE (GE Healthcare) (4/91) and Vereos PET/CT (Philips) (1/91) at approximately 66 min (95% CI: 63-68 min) post-intravenous injection of ~164 MBq (95% CI: 157-166 MBq) of ^68^Ga-DOTATATE. The majority of images (82/91) were reconstructed using ordered subset expectation maximization (OSEM) algorithm combined with time-of-flight (TOF) information and point spread function (PSF) either with 2 or 3 iterations (21 subsets). While the rest of data (9/91) were reconstructed using vendor-specific software with the recommended reconstruction parameters. Partial volume correction was not implemented.

Whole-body PET images were segmented using a semiautomatic workflow (Figure 1) based on thresholding implemented within MIM software (MIM Software Inc., Cleveland, Ohio, USA). The segmentation workflow consisted of the following steps: Organ segmentation including liver, kidneys, spleen, bone and bladder was done using an automatic workflow implemented within MIM (Contour ProtégéAI) on the attenuation correction CT of PET/CT. This workflow is consisted of a neural network framework (based on U-NET architecture) for automated contouring of normal structures on CT and MR images 13. Subsequently, the standardized uptake value (SUV) images were segmented using a thresholding technique based on a patient-specific visual inspection followed by physiological uptake removal. A visual examination was conducted on segmented mask obtained from the automated workflow to eliminate false positive and include false negative voxels.

**Figure 2:**
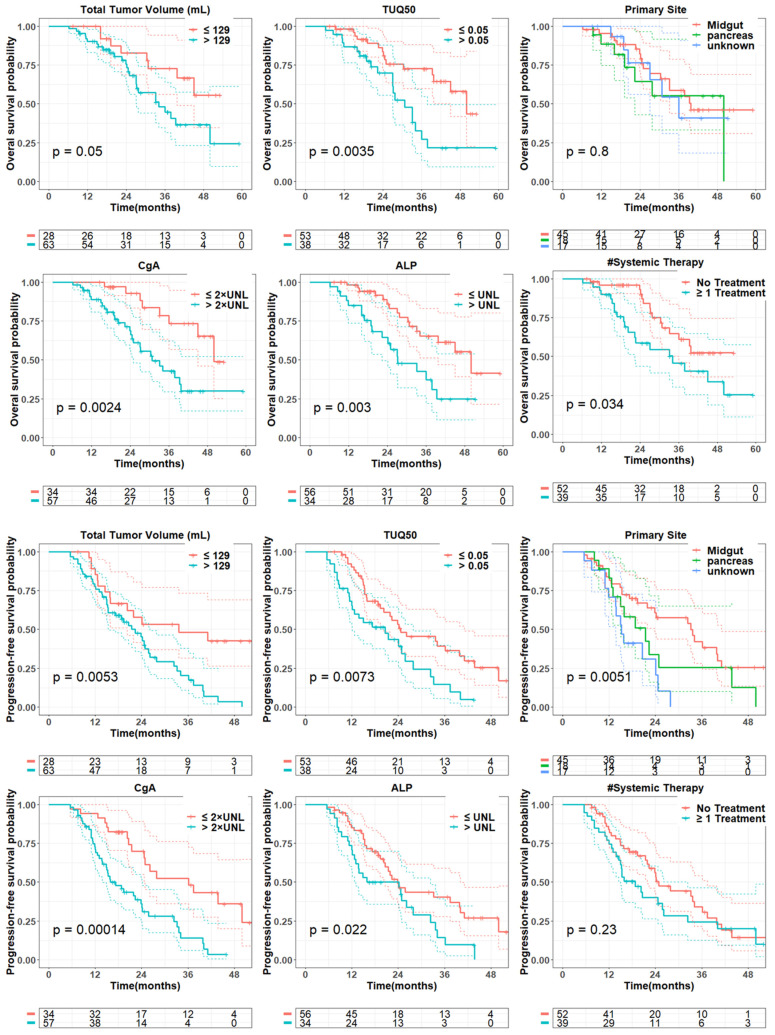
Kaplan-Meier curves for overall survival and progression-free survival based on total tumor volume, TUQ_50_, primary tumor site, ALP, CgA and number of prior systemic therapy. Tumor volumes were dichotomized into two groups based on their 30^th^ percentiles, while blood laboratory values were dichotomized based on their upper normal limit (UNL); ALP and CgA cut-off was defined UNL and 2*UNL respectively 36. Reported p-value is from log-rank test.

Each distinct lesion within the total tumor mask was annotated based on the anatomical regions (liver, bones and others). This annotation process involved aligning the anatomical masks obtained from the previous step onto the total tumor mask. Ultimately, an experienced nuclear medicine physician (KW) verified the segmentation and adjusted if needed. Examples of three cases are illustrated in Supplemental-Figure 2.

In the next step, image-based features including total and organ-specific tumor volume and SUV metrics were extracted from both the total tumor mask and each individually annotated lesion (Supplemental-Table 1). In addition, the volume of the largest tumor and the SUV of the most avid tumor were extracted. Also, the necrotic volume of liver tumor was manually outlined on pretherapy PET images, targeting the hypometabolic area within the hypermetabolic tumor tissue (classically a rim activity surrounding a central cold area), and correlated with hypodense regions in CT images. Inspired by Seifert *et al.* 14 that proposed total lesion quotient as the ratio of lesion volume to lesion SUV, we defined a metric that represents the inverse SUV of large tumors (criteria: diameter>5 cm; volume>50 mL) as tumor uptake quotient (TUQ50), based on which patients without large tumors receive a score of zero, whereas those with both large tumors and lower SUV are assigned the highest score.

TUQ50 = 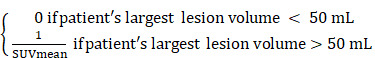


### Clinicopathological Biomarkers

A total of 23 clinical, pathologic, and laboratory biomarkers that all were hypothesized to influence tumor behavior, and treatment response were included (Supplemental-Table 1). Grade was defined based on Ki-67 or mitotic count from biopsy/surgery of primary tumor (Ki-67≤3% G1; 3% < Ki-67≤10% G2A; 10% < Ki-67≤20% G2B; Ki-67>20% G3). We further examined a metric by dividing the number of systemic treatments received prior to PRRT (excluding targeted modalities like Yttrium-90-radioembolization or external beam radiation therapy) by the time from initial diagnosis. This calculation was used to create a factor representing the growth rate of the disease in multivariate analysis.

### Statistical Analysis

OS was defined as time from the first cycle of PRRT to death from any cause. PFS was defined as time from the first cycle of PRRT to the earliest onset of progression or death from any cause. Patients lost to follow-up were censored at the date of last contact. Progression was defined as appearance of new metastatic lesions or significant increase of tumor size (not attributed to pseudo-progression) based on post-PRRT follow-up imaging that resulted in a new line of cancer treatment being commenced.

Descriptive statistics using R (version 4.3.0) software were used to examine the distribution of the exploratory features. Continuous variables with skewed distributions were log-transformed. The association between the features and the survival outcomes was assessed using univariate Cox regression models. Kaplan-Meier (K-M) curves and log-rank tests were used to compare OS and PFS between stratified groups. Features with >20% missingness were not included in multivariable analysis.

Multivariable Cox models were fit for the three sets of features: (1) PET-alone (including body weight), (2) biomarker-alone, and (3) a combined set of PET and biomarker features. Variable selection was performed using a penalized Cox regression based on least absolute shrinkage and selection operator (LASSO), where the penalty hyperparameter was tuned using nested 10-fold cross-validation. Model performance was evaluated using bootstrapped concordance index (C Index) and the Brier score based on 1000 bootstrap samples.

## Results

The analysis included 91 well-differentiated NETs (M47/F44; median age 66 years, range 34-90 years). Proliferation index (Ki-67) for 71 patients was available; G1 (n = 30), G2A/G2B (n = 22/n = 15) and G3 (n = 4). The primary tumor site was categorized as midgut (n = 45), pancreas (n = 18), unknown (n = 17) and others including bronchial, head and neck and foregut (n = 11). Four cases received reduced (50%) dose of ^177^Lu-DOTATATE in one cycle, and one patient in three cycles, because of potential risk of toxicity. Demographic information is summarized in Table 1.

The median of SSTR-avid total tumor volume was 305 mL (range 5.4-4760 mL), while the total tumor SUVmean was about 12.4 (range 2.7-43.4). 86 patients (95%) had liver metastasis with the median volume of 117.7 mL (range 0.7-4706 mL), while 64 patients (70%) showed bone metastasis with the median volume of 3.7 mL (range 0.11-325 mL).

The OS and PFS analysis accounted for 35 (38%) and 60 (66%) events throughout the study period, respectively. The median follow-up time for OS and PFS was 24.8 and 18.5 months, respectively. While the median OS time was 39.4 months (95% CI: 33.1-NA months) and the median PFS time was 23.9 months (95% CI: 19.3-32.4 months).

### Overall Survival

In univariate analysis (Table 2, Figure 2), total tumor volume was associated with worse overall survival (HR = 3.6; 95% CI: 0.92-14.2; P = 0.07). Similar findings were observed for liver tumor volume (HR = 1.7; 95% CI: 0.8-3.6; P = 0.16) and bone tumor volume (HR = 1.5; 95% CI: 1.2-2.0; P = 0.003). TUQ_50_ representing the inverse SUV of large tumors (>50 mL), was significantly associated with poor OS (HR = 1.4; 95% CI: 1.0-1.9; P = 0.03). Among the exploratory biomarkers, elevated baseline CgA (HR = 4.9; 95% CI: 1.7-13.7; P = 0.003) and ALP (HR = 52.6; 95% CI: 3.6-758.7; P = 0.004) showed a negative association with OS. Similarly, number of prior systemic treatment was associated with shorter OS (HR = 1.4; 95% CI: 1.1-1.8; P = 0.003). Also, age was a negative prognosticator (HR = 7.2; 95% CI: 0.75-69.7; P = 0.09).

In multivariate analysis (Table 3), among PET-alone features, bone tumor volume (HR = 1.6; P = 0.002), the TUQ_50_ (HR = 1.5; P = 0.008) and body weight (HR = 0.11; P = 0.02) were selected. Among biomarkers, ALP (HR = 690.5; P < 0.0001), CgA (HR = 12.4; P < 0.0001) and number of prior systemic treatment divided by time from diagnosis (HR = 1.3; P = 0.001) along with prior surgery (HR = 3.8; P = 0.002) were significant prognosticators. From combined features, only bone tumor volume (HR = 1.6; P = 0.008) was selected from image-based variables along with selected biomarkers.

### Progression Free Survival

In univariate analysis (Table 2, Figure 2), total tumor volume was associated with shorter PFS (HR, 4.3; 95% CI: 1.5-12.3; P = 0.01), Similar findings were observed for liver tumor volume (HR, 1.8; 95% CI: 1.0-3.3; P = 0.05) and bone tumor volume (HR = 1.4; 95% CI: 1.1-1.7; P = 0.01). In addition, the volume of the largest tumor (HR, 2.0; 95% CI: 1.0-3.8; P = 0.04) and TUQ_50_ (HR, 1.5; 95% CI: 1.1-1.9; P = 0.003) were significantly associated with poor PFS. Among biomarkers, elevated baseline CgA (HR = 4.2; 95% CI: 1.7-10.3; P = 0.002) and ALP (HR, 9.4; 95% CI: 1.0-92.3; P = 0.06) showed a negative association with PFS. The same was the case for number of prior systemic treatment (HR = 1.2; 95% CI: 1.0-1.5; P = 0.05). Midgut primary site of tumors versus unknown (HR = 0.3; 95% CI: 0.16-0.65; P = 0.002) and pancreas primary site versus unknown (HR = 0.5; 95% CI: 0.2-1.2; P = 0.11) showed a longer PFS.

**Figure 3:**
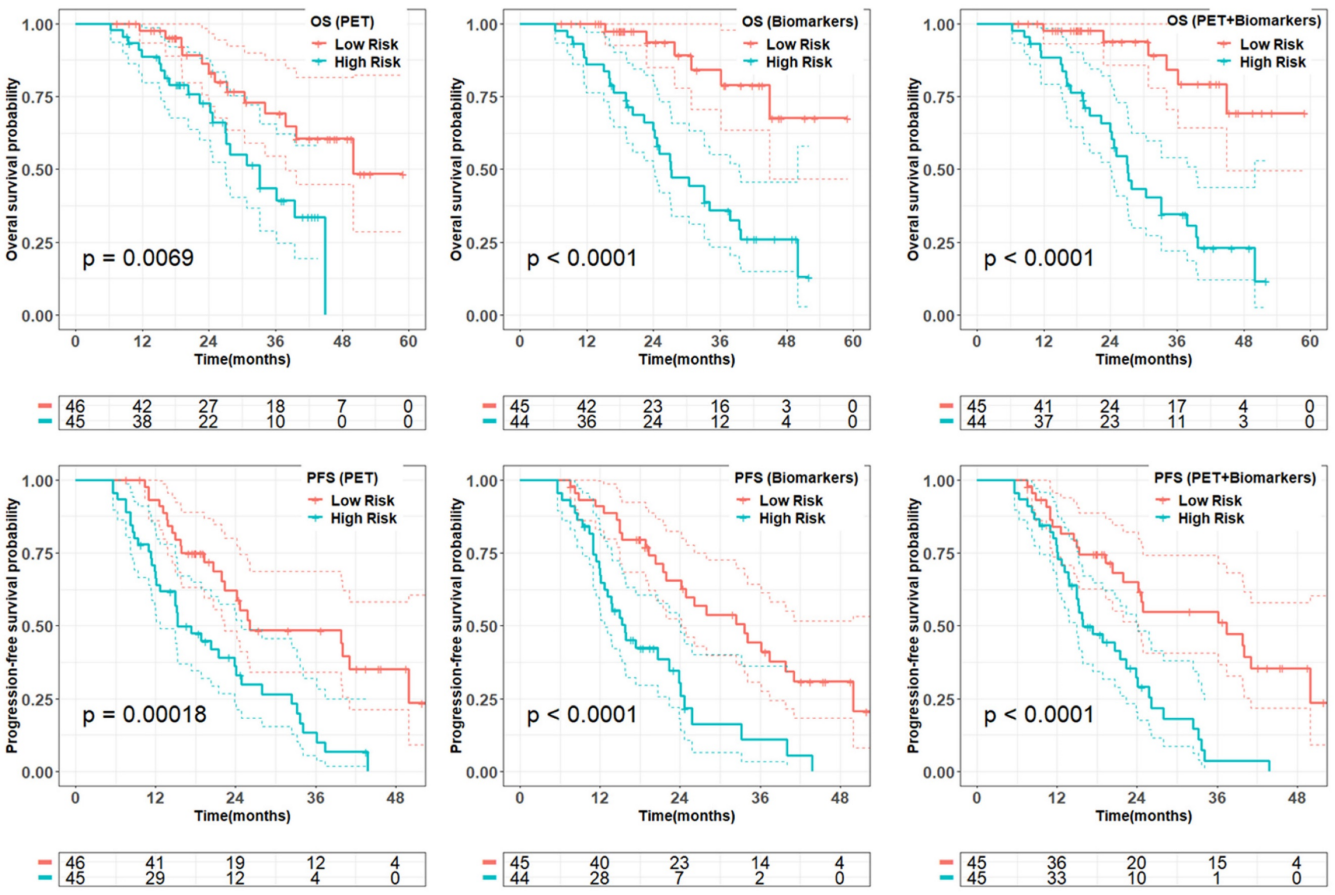
Quality of the prognostic and predictive risk scores from multivariate models (Table 3) on PET-alone, biomarkers-alone, and combined model in the whole data set. Patients with the predicted risk score greater than the median of the total cohort was dichotomized as high risk. Reported p-value is from log-rank test.

From penalized multivariate analysis (Table 3), among PET features, liver tumor SUVmean (HR = 1.9; P = 0.04), bone tumor volume (HR = 1.4; P = 0.002) along with TUQ_50_ (HR = 1.5; P = 0.002) were selected. Among biomarkers, CgA (HR = 7.1; P = 0.0001), ALP (HR = 4.9; P = 0.2), number of prior systemic treatment divided by time from diagnosis (HR = 1.2; P = 0.1) and primary tumor site (HR = 1.3; P = 0.007) were significant predictive features. Patients with risk score greater than the median predicted from the multivariable Cox models (Table 3), were plotted using Kaplan-Meier estimator in Figure 3.

We evaluated the accuracy of the proposed multivariate models through 1000 bootstrap analysis summarized in Table 4. We further evaluated the performance of the whole process including penalized algorithm for feature selection followed by Cox regression in a bootstrap analysis. The biomarker model demonstrated prognostic accuracy, as measured by a C index and Brier score, of 0.77 ± 0.09 and 0.10 ± 0.03 while combined model slightly improved the model performance. The PET and biomarker model showed similar accuracy in prediction of PFS with C Index of 0.63 ± 0.09 and 0.66 ± 0.09, respectively. However, the incorporation of combined features into penalized model did not enhance the overall model performance.

## Discussion

^177^Lu-PRRT has shown promising potential as an effective targeted therapy in well-differentiated metastatic NETs by improving quality of life and prolongation of PFS, however the specific response to PRRT can vary, and not all patients experience the same level of benefit. Hence, the development of a robust patient selection criteria rather than simplified qualitative metrics is imperative 15. To address this unmet need, we evaluated the prognostic and predictive value of SSTR-PET features along with a comprehensive set of clinicopathological biomarkers on PRRT outcome.

In NETTER-1 trial, among 110 well-differentiated midgut NETs the median OS was reported to be 48 months and the PFS at 20 months was found to be 65.2% for the cohort receiving PRRT. Our study showed a shorter OS of 39.4 months and a comparable PFS at 20 months of 59%. We observed a median PFS of 23.9 months which is shorter than the PFS reported from NETTER-1. However, our subgroup analysis of midgut NETs (n = 45) showed a median PFS of 33.2 months 4, 16.

We investigated the association of pretherapy SSTR-PET quantitative metrics with PRRT outcome. Several studies have correlated higher SSTR density quantified by baseline ^68^Ga-PET with improved PRRT outcome 17, 18. In our study, SSTR density in terms of total tumor SUVmean was not correlated with outcome (P>0.48). However, the TUQ_50_ representing the inverse SUV of the largest tumor (> 50 mL) was significantly associated with worse PFS (HR = 1.5; P = 0.003) and OS (HR = 1.4; P = 0.03), implying that higher SSTR density is associated with a more favorable PRRT outcome (Figure 2: TUQ_50_ cutoff of 0.05 is equal to SUV of 20). Notably, unlike FDG PET, where total tumor glycolysis (TLG = tumor volume × tumor SUV) is associated with tumor proliferation and worse outcomes, total tumor SSTR expression consists of total tumor volume, a negative predictor of survival, and tumor SUV, a positive predictor, opposing effects may negate a predictive value of SSTR density 14. Therefore, tumor uptake quotient was found to be significantly associated with poor survival.

SSTR-positive tumor volume has been reported as a factor of worse prognosis and predictive of PFS 9, 19. We identified the total tumor volume was associated with poor PFS (HR = 4.3; p = 0.01) and OS (HR = 3.6; p = 0.07). Also, liver tumor volume as the dominant site of distant metastasis amongst NETs was shown to be a poor prognostic factor 20, 21. However, two previous studies demonstrated no difference in PFS based upon the extent of hepatic tumor burden 22, 23. We observed a negative association of liver tumor volume with both PFS (HR = 1.8; P = 0.05), and OS (HR = 1.7; P = 0.16), however, it was not statistically significant. We considered that the typically larger size of liver tumors compared to tumors in other organs contributes to the strong correlation between liver tumor volume and total tumor volume. In our study, we found a strong correlation between total tumor volume and liver tumor volume (ρ = 0.9; P < 0.001). This implies that the overall extent of the disease (total tumor volume) is negatively associated with the outcome, not specifically hepatic involvement. Of note, liver tumor burden as the percentage of tumor volume within the liver was associated with poor PFS, but not significantly (HR = 1.1; P = 0.23). This implies, unlike surgical procedures, that disease burden is a critical factor representing preserved healthy tissue, and that in PRRT, the percentage of liver involvement is not a significant predictor, given that PRRT is well-tolerated and does not cause serious hepatic toxicity. Also, necrotic volumes of liver tumors identified in ^68^Ga-PET, present in 18 (20%) cases, demonstrated a poor prognostic value, suggesting that tumor necrosis may indicate a more aggressive tumor biology 24.

Previous studies have shown that the presence of bone metastases in NETs is linked to a shorter OS 20, 25. In our analysis, an elevated bone tumor volume was correlated with poor outcome (HR = 1.5; p = 0.003). Sabet *et al.* 26 demonstrated that bone metastasis can be effectively controlled by PRRT (mainly because of their small size), however the presence of extended bone tumors is associated with more advanced stage of disease and therefore associated with shorter PFS. We observed that total bone tumor volume larger than 3 mL that is roughly associated with an average of five focal bone metastasis is associated with worse outcome, concordant with findings reported by Swiha *et al.* 20.

The size of the tumor is known to be correlated with the therapeutic effectiveness of radiolabeled targeted therapy, particularly in short-range radionuclides such as ^177^Lu (beta/gamma emitter, average beta range in soft tissue ~ 0.23 mm) 27, 28. A post hoc analysis of NETTER-1 demonstrated that the absence of a large tumor (diameter > 30 mm) was associated with improved PFS 23. We evaluated the impact of tumor size in terms of SSTR-positive largest tumor volume on PFS that showed a significant association with shorter PFS (HR = 2.0, P = 0.04).

Several studies have investigated non-imaging biomarkers of NETs that affect PRRT outcome. Ezziddin *et al.* demonstrated Ki-67 > 10% is a poor prognostic and predictive factor 21, 26. Their finding was confirmed by Albersberg *et al.* 3 showing the predictive value of Ki-67 of more than 5% for PFS and more than 10% for OS. Thuillier *et al.* 19 showed Ki-67 > 20% (G3) is a negative prognostic factor. However, in the current study we did not observe a significant impact of tumor histologic parameter (Ki-67) with OS (HR = 1.2; P = 0.3) and PFS (HR = 1.3; P = 0.11). This observation might be influenced by the high number of missing values (22%) which were generally due to remote pathology specimens where Ki-67 reporting was not routinely performed. Also, we observed four cases with Ki-67>20% that demonstrated shorter median OS (21 months) and PFS (11 months).

Previous studies have documented longer survival for midgut-NETs compared to pancreas, lung and unknown origin tumors, possibly owing to a more indolent nature of midgut-NETs 20, 21, 29. In our study, median PFS for midgut tumors was 34 months versus 21 months for pancreatic and 16 months for unknown (P = 0.005). This might be due to the more aggressive type of pancreas-originated tumors compared to midgut tumors and the more advanced stage of unknown primary neuroendocrine tumors at the time of diagnosis. However, we did not observe any significant prognostic value based on the primary tumor site (Figure 2).

There is not a firm conclusion regarding the impact of prior systemic treatment on the effectiveness of PRRT 22, 30. In our study, the number of prior systemic treatment was associated with shorter survival (HR = 1.4; P = 0.003). Prior systemic treatment highlights the progression of disease and its resistance against previous lines of therapy. We divided the number of prior systemic treatment by the time from diagnosis in order to create a factor representing the growth rate of the disease; this factor was significantly associated with poor OS (HR = 1.6; CI = 1.1-2.2, P = 0.02) and PFS (HR = 1.5; CI = 1.0-2.2, P = 0.05).

Among available baseline blood biomarkers, elevated Chromogranin A was found a strong negative predictor of survival (OS: HR = 4.9; P = 0.003; PFS: HR = 4.2; P = 0.002) which is consistent with other studies including the RADIANT trial 12, 20, 23, 31. While changes in CgA can indicate treatment response if declining, or conversely progression of disease if rising, there are debates regarding the role of CgA as a prognostic biomarker because of its high false-positivity (related to proton pump inhibitor, kidney failure, and heart disease) and false-negativity (non-secretory tumors) 32.

Elevated Alkaline Phosphatase which may be linked with extensive liver and bone metastases has been reported a poor predictive factor associated with PRRT survival 31, 33, 34. In our study, ALP showed a strong prognostic value (HR = 52.6; P = 0.004) and moderate predictive value (HR = 9.4; P = 0.06). Nevertheless, in the post hoc analysis of the NETTER-1 trial, ALP was not identified as being associated with PFS 23.

In addition, we investigated the association of some inflammatory factors as platelet-lymphocyte ratio and neutrophil-lymphocyte ratio with survival 35. We did not observe a significant correlation between neutrophil- lymphocyte ratio (HR = 1.1; P = 0.64) and platelet-lymphocyte ratio (HR = 2.6; P = 0.57) with OS. As it is known, age is associated with shorter OS, however, PFS was not influenced by patient age in our population. Also, patient weight was selected in multivariate models including PET features that might be due to its influence on the distribution of radiopharmaceuticals and the SUV quantitative metrics.

One strength of our study lies in the diversity and heterogeneity within our NET cohort that may reflect a realistic clinical practice of PRRT. Another advantage of this study is the precise quantification of SSTR-PET metrics, achieved through a meticulous manual verification of tumor segmentation. Moreover, this study is the first investigation exploring a comprehensive set of image-based features and biomarkers in relation to PRRT outcomes. However, it bears some limitations. The analysis is based on single-center database and was performed retrospectively and therefore is susceptible to selection biases. The limited size of the patient cohort could potentially impact generalizability. Furthermore, the patients in our analysis had a restricted follow-up time post-PRRT, leading to a significant number of censoring events. Lastly, blood-based genomic biomarkers and FDG-PET, which have been reported as prognostic/predictive factors, were not included in the analysis because they are not part of routine clinical protocol at our center.

## Conclusion

In summary, we demonstrated that image-based features including SSTR-avid tumor volume, bone lesion involvement and the presence of large tumors as well as elevated baseline chromogranin A and alkaline phosphatase along with the prior systemic treatments have negative correlation with PRRT PFS and OS. These findings could inform pre-therapy assessments, guiding PRRT treatment decisions and patient stratification in future trials.

## Supplementary Material

Supplementary figures and table.

## Figures and Tables

**Table 1:** Patient demographic information.

Characteristic	(n = 91)
Sex	
Female / Male	44 (48%) / 47 (52%)
Age at first treatment - median (range)	66 (34-90)
Primary tumor site	
Midgut (small bowel, ileum, jejunum, colon)	45 (48%)
Pancreatic	18 (20%)
Unknown	17 (19%)
Other (bronchial, foregut)	11 (12%)
Tumor grade	
G1	30 (33%)
G2A/ G2B	22 (24%) / 15 (17%)
G3	4 (4%)
Missing	20 (22%)
Prior treatments	
Surgery	54 (59%)
Systemic treatment (≥1)	39 (43%)
Liver directed treatment or 90Y-SIRT	34 (37%)
Blood biomarkers	
CgA (IU/L)	309 (20-1.9e4)
ALP (ng/mL)	106 (27-530)
Tumor volume	
Total tumor volume (mL)	305.9 (5.4-4760)
Liver tumor volume (mL)	95.4 (0.0-4760)
Bone tumor volume (mL)	1.6 (0.0-2049)

**Table 2:**
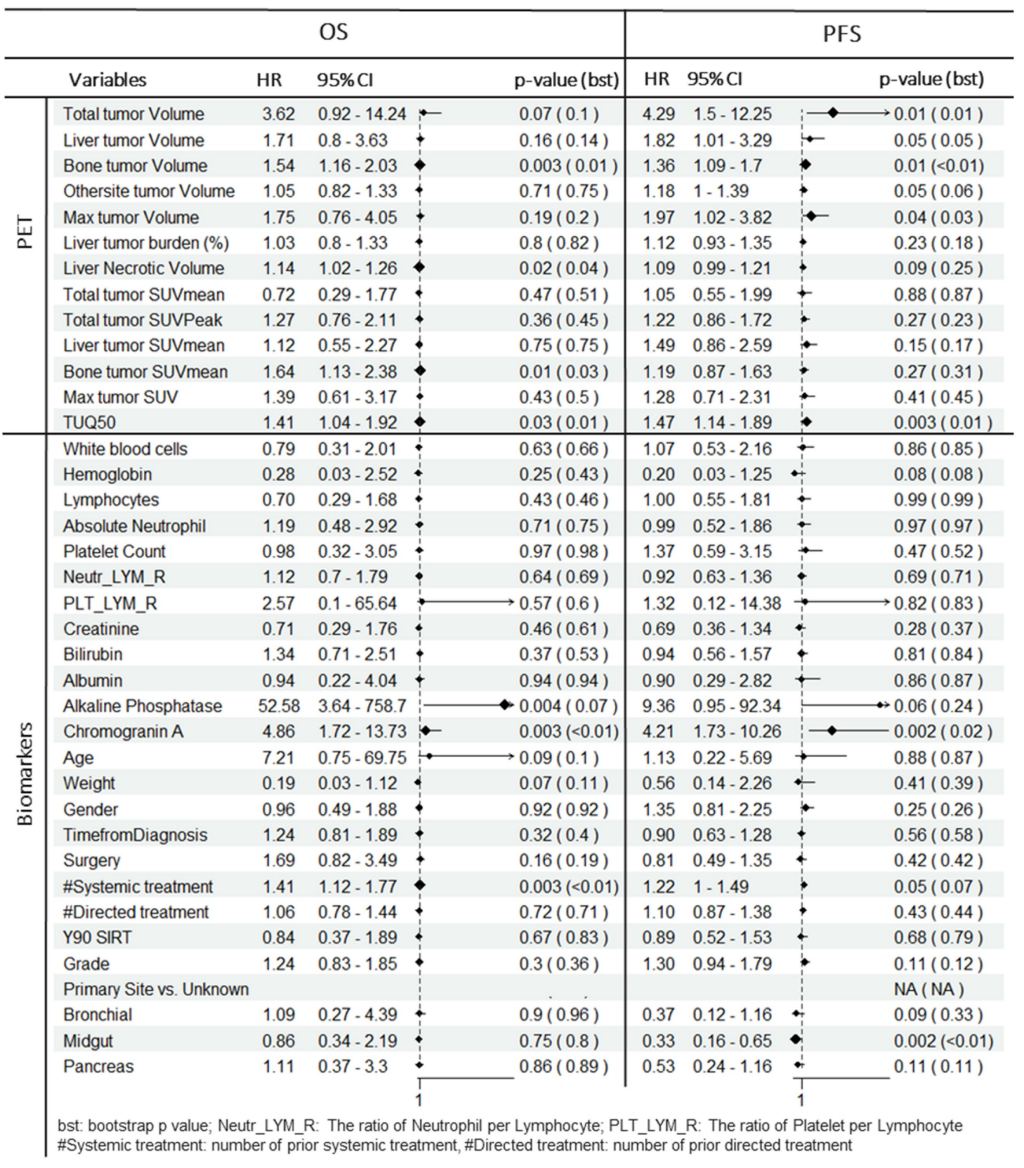
Univariate Cox regression analysis for OS and PFS. Tumor volumes, CgA and ALP are log transformed. Each variable is normalized by its mean. Bootstrap p value is presented along with original p value to robustly evaluate the statistical significance of covariate effects.

**Table 3:** Selected features from multivariable penalized Cox regression algorithm (LASSO). Tumor volumes, CgA and ALP are log transformed. Each variable is normalized by its mean.

Survival	Features	Predictors	PET-alone / Biomarkers-alone			Combined (PET+Bio)		
			HR	95% CI	p value	HR	95% CI	p value
OS	PET	Bone tumor volume	1.6	1.2-2.2	0.002	1.6	1.1-2.1	0.008
		TUQ_50_	1.5	1.1-2.0	0.008			
		Weight	0.11	0.0-0.7	0.02			
	Bio	CgA	12.4	3.7-41.3	< 0.0001	9.7	2.9-32.9	< 0.0001
		ALP	690.5	26.3-1e4	< 0.0001	879.7	39.6-1e4	< 0.0001
		Surgery	3.8	1.6-9.0	0.002	2.7	1.2-6.5	0.02
		#Systemic treatment/Time from diagnostic	1.34	1.1-1.6	0.001	1.2	1.0-1.5	0.02
PFS	PET	Liver tumor SUVmean	1.9	1.0-3.5	0.04	2.30	1.2-4.4	0.01
		Bone tumor volume	1.4	1.1-1.8	0.002	1.3	1.1-1.7	0.02
		TUQ_50_	1.5	1.2-2.0	0.002	1.3	1.0-1.8	0.06
		Weight	0.38	0.09-1.6	0.19	0.3	0.1-1.1	0.07
	Bio	CgA	7.1	2.7-18.4	0.0001	4.3	1.6-11.3	0.003
		ALP	4.9	0.43-56.1	0.2			
		Primary Site	1.3	1.07-1.5	0.007	1.2	1.0-1.5	0.02
		#Systemic treatment/Time from diagnostic	1.2	0.97-1.4	0.10	1.3	1.1-1.6	0.004

**Table 4:** Model performance validation using selected features through bootstrap analysis. Whole process validation representing the model performance of feature selection plus Cox regression through bootstrap samples.

		OS				PFS			
Variable set		C Index		Brier		C Index		Brier	
		mean	S.E.	mean	S.E.	mean	S.E.	mean	S.E.
**Model performance with selected features**									
PET		0.68	0.11	0.12	0.04	0.63	0.09	0.17	0.03
Bio		0.77	0.09	0.10	0.03	0.66	0.09	0.16	0.03
PET + Bio		0.78	0.09	0.10	0.03	0.66	0.09	0.15	0.03
**Model performance including variable selection**									
PET		0.59	0.20	0.12	0.07	0.62	0.15	0.17	0.05
Bio		0.72	0.19	0.09	0.06	0.60	0.15	0.17	0.05
PET + Bio		0.71	0.19	0.1	0.06	0.57	0.14	0.18	0.05
