# Supplementary material for: Enhancing precision: A predictive model for 177Lu-DOTATATE treatment response in neuroendocrine tumors using quantitative 68Ga-DOTATATE PET and clinicopathological biomarkers
Source: Theranostics. 2024 Jun 11;14(9):3708–18. doi: 10.7150/thno.98053 (PMC11209719; doi:10.7150/thno.98053)
Supplement: Supplementary file 1 — Supplementary figures and table. [file thnov14p3708s1.pdf]

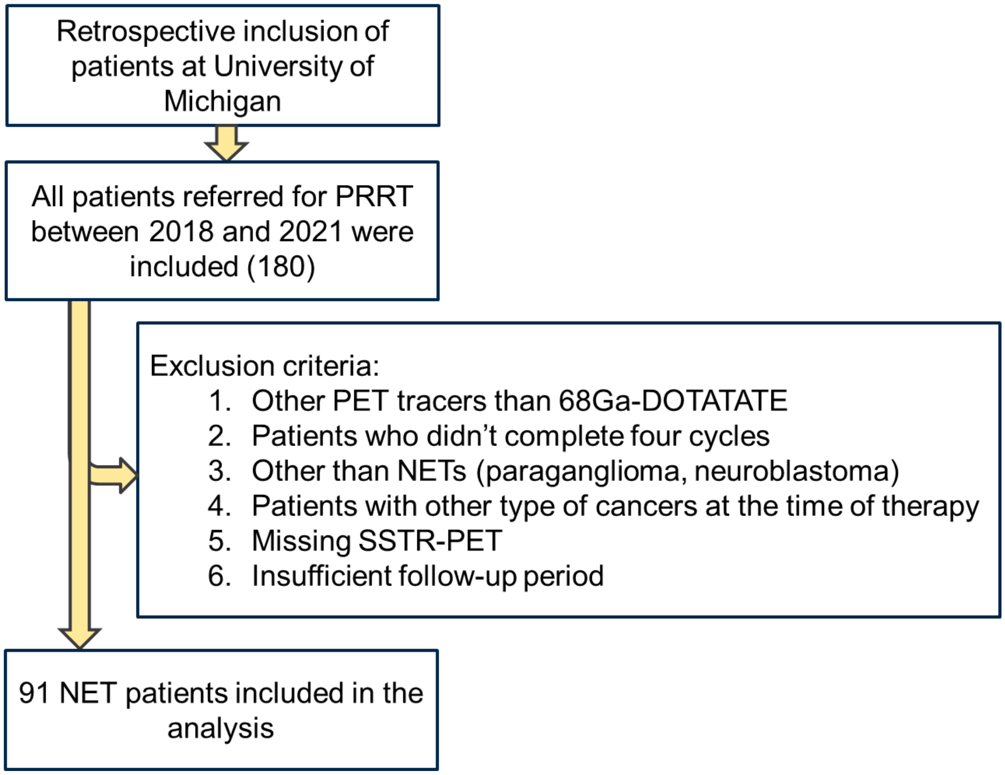

Supplemental-Figure 1. We evaluated a cohort of 180 patients who received at least one cycle of ^{177}Lu-Dotatate in a routine clinical setting between 2018 and 2021 at the University of Michigan. This study was approved by the local Institutional Review Board and informed consent was obtained from participants. We excluded 89 patients according to the following criteria: (1) use of PET tracer other than ^{68}Ga-DOTATATE; (2) those who did not complete four cycles; (3) other tumors (paraganglioma, neuroblastoma); (4) patients with other type of cancers at the time of therapy; (5) missing SSTR-PET; (6) insufficient follow-up period. 91 patients were included for OS analysis. At the time of data lock (September 2023), 35 patients (38%) had passed away, while 60 patients (66%) had experienced progression.

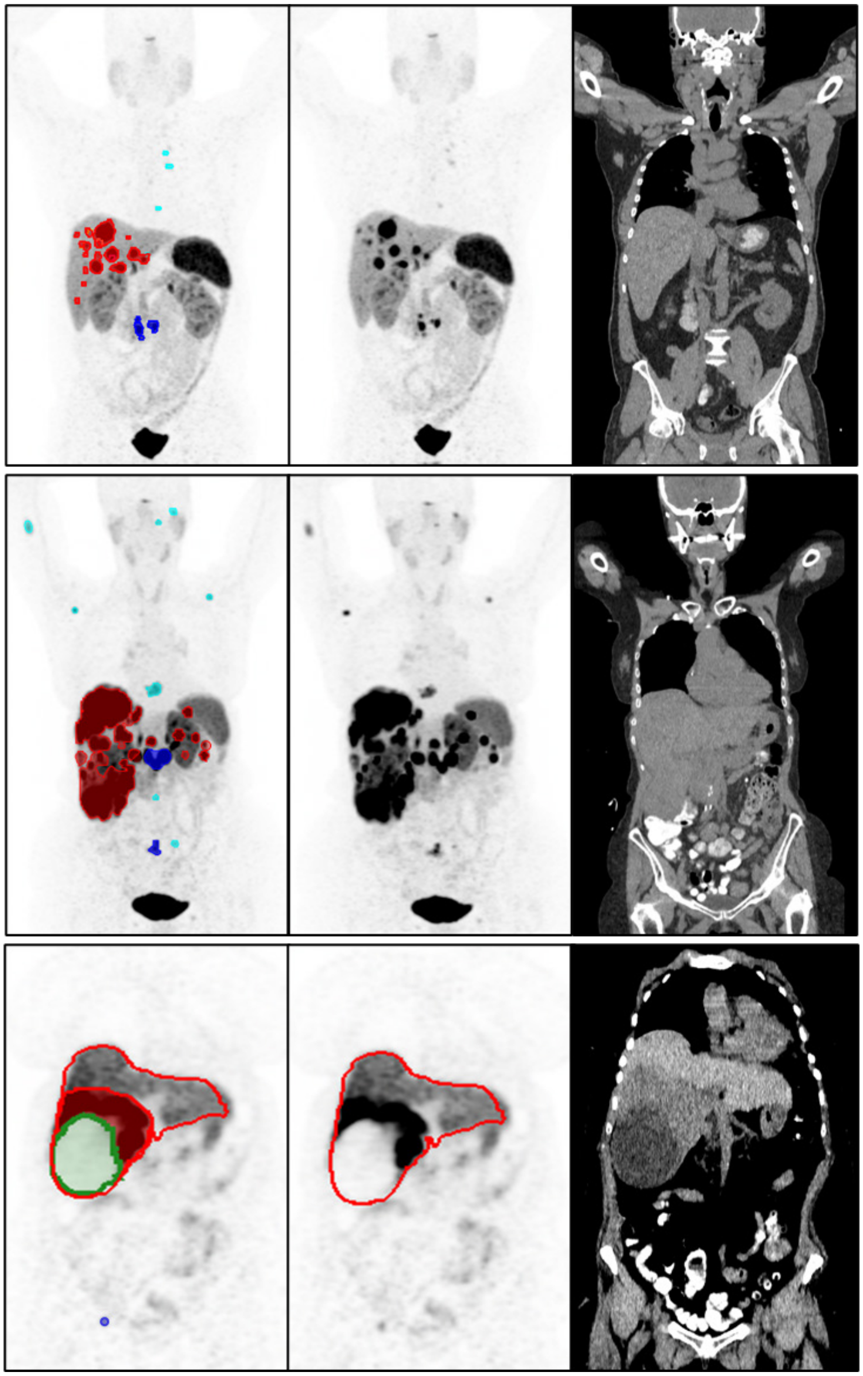

Supplemental-Figure 2. Representative examples of the segmentations from our semi-automatic workflow for three patients. Maximum intensity projection of ^{68}Ga-PET with tumor segmentation (left panel; red: liver tumors, cyan: bone tumors, dark blue: other tumors) without tumor mask (middle) and CT images (right). The bottom panel demonstrates a patient with liver necrotic volume. PET SUV ranges from 0 to 10; Hounsfield unit (HU) ranges -100 to 200; The bottom CT-HU ranges between -40 and 100.

Supplemental-Table 1. Complete variable set, including PET and clinicopathological features, used in the statistical analysis.

	Name of feature	Description
PET	Total Lesion Volume	Total tumor volume (mL)
	Total Tumor SUVmean	Average SUV of the entire total tumor volume
	Total Tumor SUVPeak	Average SUV within a 1 cm^3 sphere centered on the site of highest uptake in the entire total tumor volume
	Liver Tumor Volume	Total tumor volume in the liver (mL)
	Liver Tumor burden	Total tumor volume in the liver divided by the liver volume (%)
	Liver Necrotic Volume	Tumor necrotic volume in the liver (mL)
	Liver Tumor SUVmean	Average SUV of the entire liver tumors
	Bone Tumor Volume	Total tumor volume in bones (mL)
	Bone Tumor SUVmean	Average SUV of the entire bone tumors
	Othersite Tumor Volume	Total tumor volume in organs other than liver and bone (mL)
	Max Tumor Volume	Volume of the largest tumor within the patient (mL)
	Max Tumor SUV	Average SUV of the hottest tumor within the patient
	TUQ50	Inverse SUVmean of the largest tumor with volume larger than 50 mL, 5 cm in diameter
Biomarkers	Time from Diagnosis	Time between the first diagnosis to the first cycle of PRRT
	Grade	Histologic grade (using Ki-67 index) of primary tumor from biopsy/surgery
	Primary tumor site	Primary tumor site
	#Systemic therapy	Number of prior systemic treatments (chemotherapy or other)
	#Directed therapy	Number of prior liver directed treatments (TACE, Y90, cryotherapy)
	Y90 SIRT	Prior liver directed treatment with Y90-SIRT
	Surgery	Tumor resection
	White blood cells	White Blood Cells (K/cmm)
	Hemoglobin	Hemoglobin (g/dL)
	Lymphocytes	Lymphocytes (K/uL)
	Absolute Neutrophil	Absolute Neutrophil Count (K/cmm)
	Platelet	Platelet Count (K/cmm)
	PLT_LYM_R	The ratio of Platelet to Lymphocytes
	Neutr_LYM_R	The ratio of Absolute Neutrophil Count to Lymphocytes
	Creatinine	Creatinine (mg/dL)
	Bilirubin	Bilirubin (mg/dL)
	Albumin	Albumin (mg/dL)
	ALP	Alkaline Phosphatase (IU/L)
	CgA	Chromogranin A (Tumor Marker) (ng/mL)
